# Supplementary material for: Serum Neurofilament Light Chain as Biomarker for Cladribine-Treated Multiple Sclerosis Patients in a Real-World Setting
Source: Int J Mol Sci. 2023 Feb 17;24(4):4067. doi: 10.3390/ijms24044067 (PMC9961994; doi:10.3390/ijms24044067)
Supplement: Supplementary file 1 [file ijms-24-04067-s001.zip › ijms-2138396-supplementary.pdf]

Suppl. Figure S1. Disease course and sNfL of the individual patients (n = 14) receiving CLAD therapy.

- NEDA-3 achieved

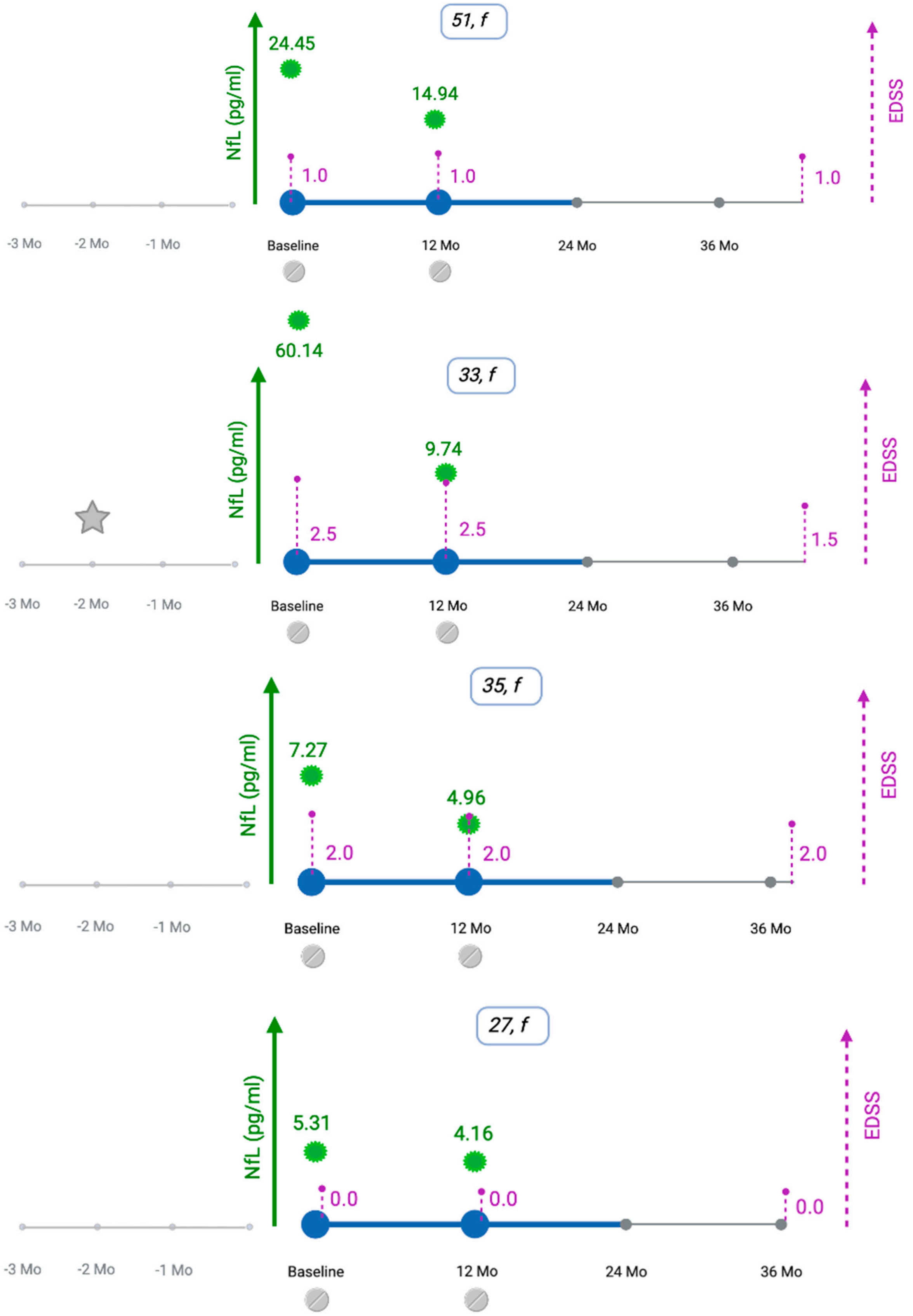

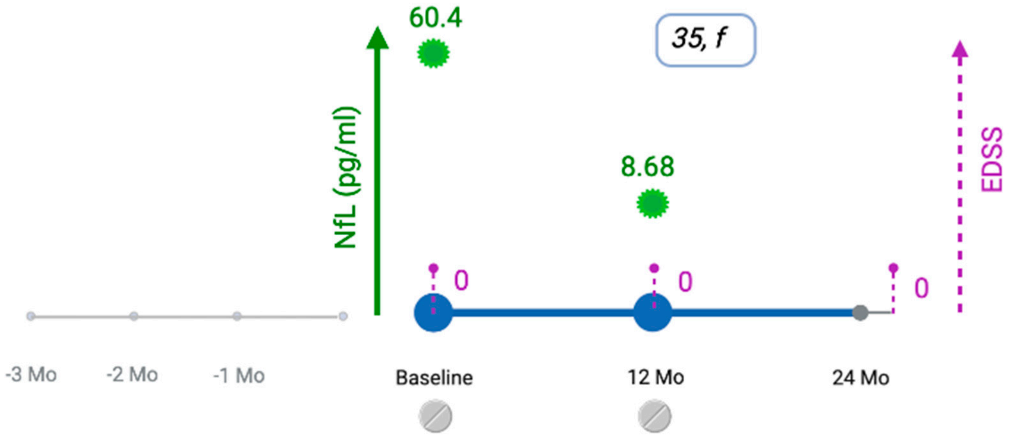
60.4
35, f
NfL (pg/ml)
8.68
EDSS
0
0
0
-3 Mo
-2 Mo
-1 Mo
Baseline
12 Mo
24 Mo

- No NEDA-3:

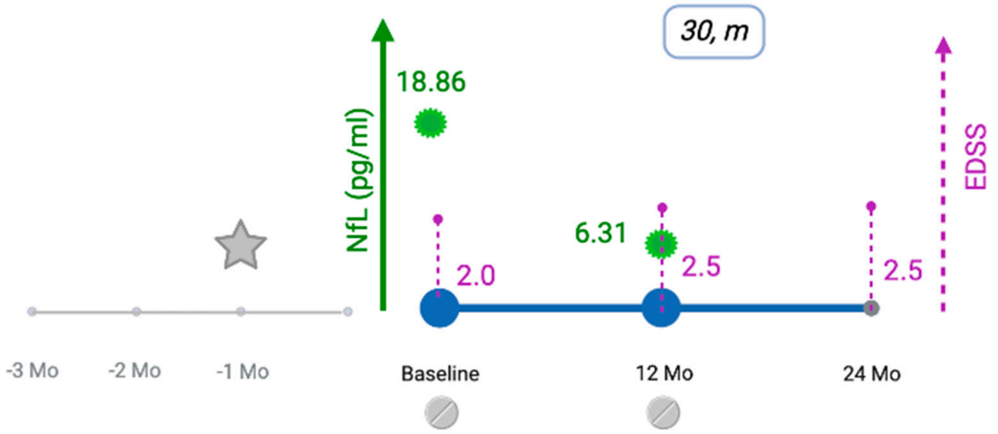
30, m
18.86
NfL (pg/ml)
EDSS
6.31
2.0
2.5
2.5
-3 Mo
-2 Mo
-1 Mo
Baseline
12 Mo
24 Mo

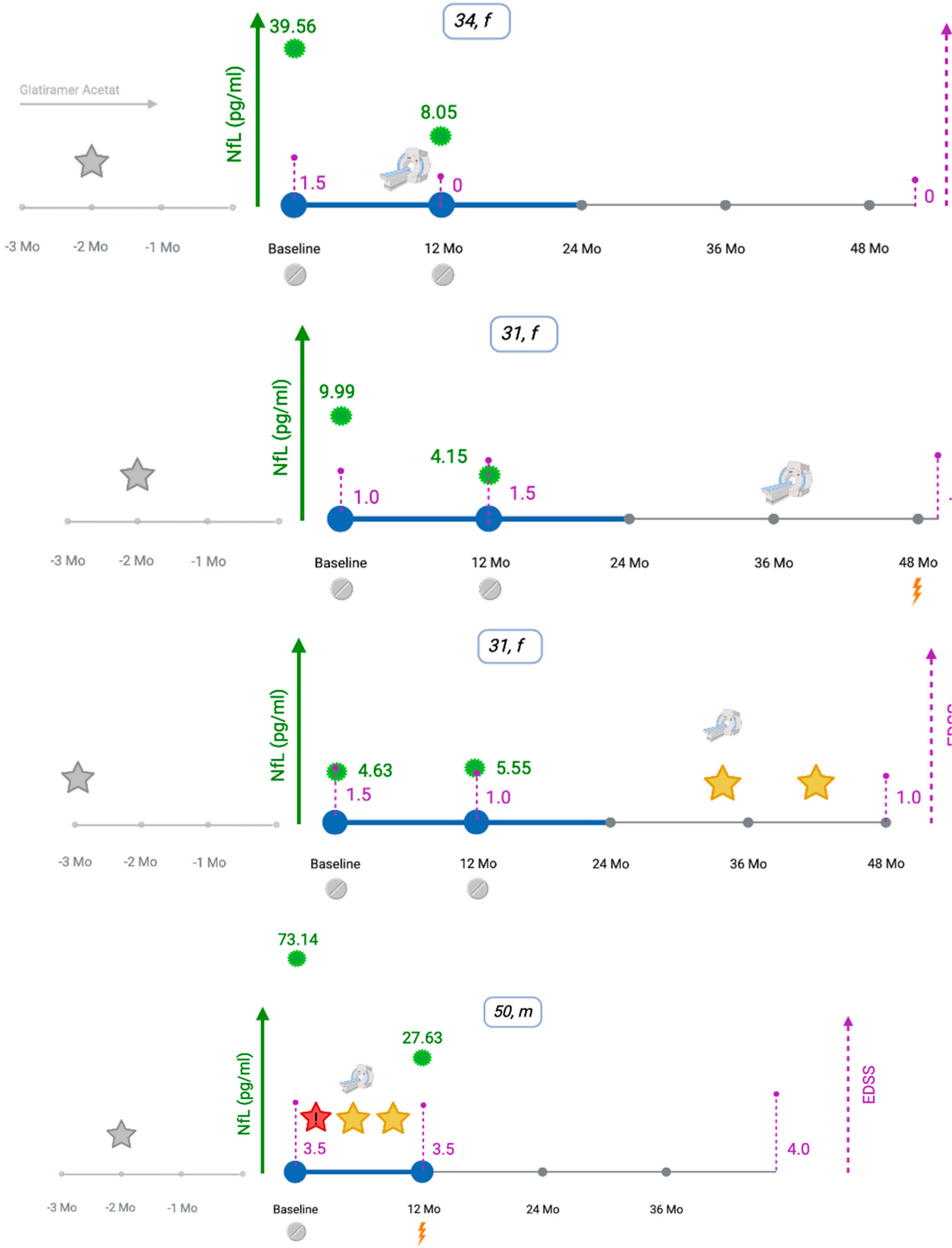
34, f
Glatiramer Acetat
NfL (pg/ml)
39.56
8.05
1.5
0
0
-3 Mo
-2 Mo
-1 Mo
Baseline
12 Mo
24 Mo
36 Mo
48 Mo
31, f
NfL (pg/ml)
9.99
4.15
1.0
1.5
-3 Mo
-2 Mo
-1 Mo
Baseline
12 Mo
24 Mo
36 Mo
48 Mo
31, f
NfL (pg/ml)
4.63
5.55
1.5
1.0
1.0
-3 Mo
-2 Mo
-1 Mo
Baseline
12 Mo
24 Mo
36 Mo
48 Mo
73.14
50, m
NfL (pg/ml)
27.63
3.5
3.5
4.0
EDSS
-3 Mo
-2 Mo
-1 Mo
Baseline
12 Mo
24 Mo
36 Mo

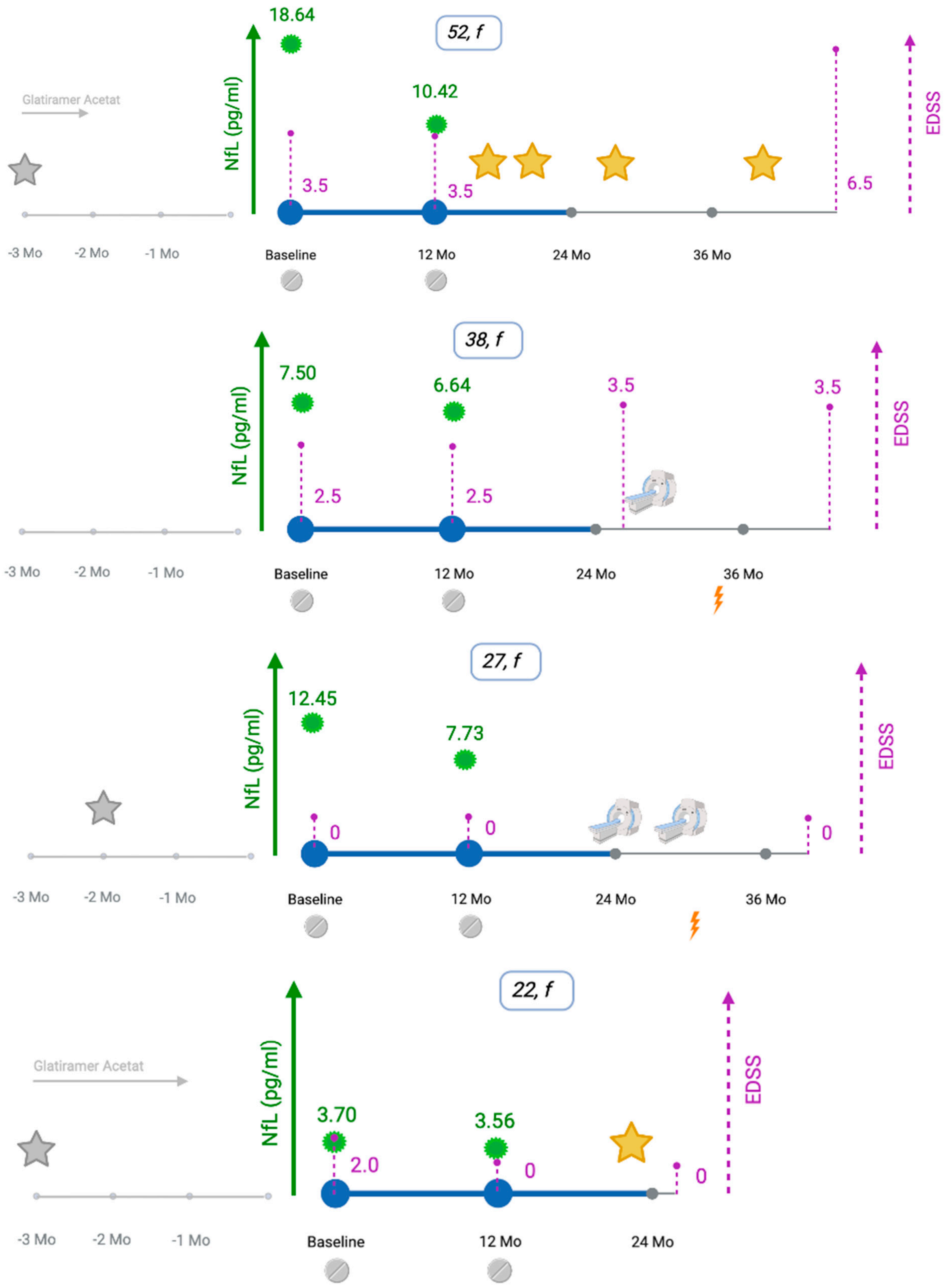

18.64
52, f
Glatiramer Acetat
NfL (pg/ml)
10.42
3.5
3.5
6.5
EDSS
-3 Mo
-2 Mo
-1 Mo
Baseline
12 Mo
24 Mo
36 Mo
38, f
7.50
6.64
3.5
3.5
NfL (pg/ml)
EDSS
2.5
2.5
-3 Mo
-2 Mo
-1 Mo
Baseline
12 Mo
24 Mo
36 Mo
27, f
12.45
7.73
NfL (pg/ml)
EDSS
0
0
0
-3 Mo
-2 Mo
-1 Mo
Baseline
12 Mo
24 Mo
36 Mo
22, f
Glatiramer Acetat
NfL (pg/ml)
EDSS
3.70
3.56
2.0
0
0
-3 Mo
-2 Mo
-1 Mo
Baseline
12 Mo
24 Mo

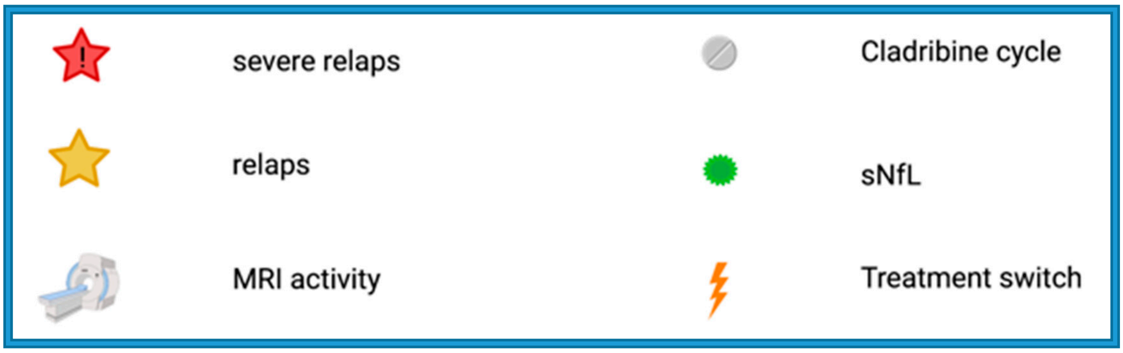

NEDA: no evidence of disease activity
